# Third Edition of Dr. Harris’ Work on Dentistry

**Published:** 1848-01

**Authors:** 


### THIRD EDITION OF DR. HARRIS' WORK ON DENTISTRY.

We had prepared a notice of this excellent work, the third edition of which is now from the press, and, with the alterations and additions, make a work of some seven hundred and fifty pages. For the want of room, this, with several communications, as well as a short essay on the effects of *Calomel* on the Teeth, (which was promised in the first No. of the Register by ourself,) cannot appear until the next number.

Two or three cases reported by Dr. Berry of this city, now in Mississippi, and also the description of a very convenient and cheap furnace by Dr. Hoy, of Arkansas, was mislaid during our illness, and as yet, have not come to light. We hope our friends will re-furnish their communications, so that, should they not be found, they may still appear in the next.

Cases of Malformation, by Dr. Smith, will also appear in our next number.—Cin. Ed.

